# Voltage controlled core reversal of fixed magnetic skyrmions without a magnetic field

**DOI:** 10.1038/srep31272

**Published:** 2016-08-10

**Authors:** Dhritiman Bhattacharya, Md Mamun Al-Rashid, Jayasimha Atulasimha

**Affiliations:** 1 Department of Mechanical and Nuclear Engineering, Virginia Commonwealth University, Richmond, VA 23284, USA; 2 Department of Electrical and Computer Engineering, Virginia Commonwealth University, Richmond, VA 23284, USA

## Abstract

Using micromagnetic simulations we demonstrate core reversal of a fixed magnetic skyrmion by modulating the perpendicular magnetic anisotropy of a nanomagnet with an electric field. We can switch reversibly between two skyrmion states and two ferromagnetic states, i.e. skyrmion states with the magnetization of the core pointing down/up and periphery pointing up/down, and ferromagnetic states with magnetization pointing up/down, by sequential increase and decrease of the perpendicular magnetic anisotropy. The switching between these states is explained by the fact that the spin texture corresponding to each of these stable states minimizes the sum of the magnetic anisotropy, demagnetization, Dzyaloshinskii-Moriya interaction (DMI) and exchange energies. This could lead to the possibility of energy efficient nanomagnetic memory and logic devices implemented with fixed skyrmions without using a magnetic field and without moving skyrmions with a current.

The ongoing quest for high density and high speed nanomagnetic computing devices has led to the exploration of novel materials, devices and switching strategies. Recently, a topologically protected spiral spin structure called skyrmion has attracted attention due to its potential use as such devices. Skyrmions were first proposed to explain hadrons^1^. Later theories predicted the existence of magnetic skyrmions in the chiral helimagnets^2^,^3^. Subsequently, experiments showed the evidence of skyrmion lattices in bulk^4^,^5^,^6^ and thin film^7^,^8^,^9^,^10^. Here Dzyaloshinskii-Moriya interaction (DMI)^11^,^12^, given by 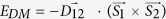 (where 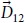 is the DMI vector, 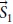 and 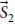 are two atomic spins) that is present in non-centrosymmetric magnets or thin films interfaced with a metal with large spin orbit coupling, stabilizes the skyrmion state. Figure 1(a) shows a schematic of the magnetic configuration of a skyrmion. Several schemes have been investigated to design racetrack memories^13^,^14^,^15^ and logic gates^16^ by manipulating the motion of a skyrmion because the pinning current is orders of magnitude less than that of domain walls^17^,^18^. Although an ultra-dense memory device with readout integrated to a magnetic tunnel junction (MTJ) can be assembled via core reversal of stationary skyrmion, such *in-situ* control of magnetic state of skyrmions has not been studied extensively. Core reversal induced by microwave^19^, magnetic field^20^, spin current^21^, and conversion between Skyrmion and ferromagnetic state using Scanning Tunnelling Microscope tip^22^ and combination of electrical and magnetic fields^23^ have been shown. However, skyrmion core reversal with voltage controlled magnetic anisotropy has only been shown by Nakatani *et al*.^24^ and our group so far. This paper proposes an extremely energy efficient route for core reversal of a magnetic skyrmion with an electric field.

Previous schemes for switching nanomagnets with perpendicular magnetic anisotropy (PMA) using voltage control of magnetic anisotropy (VCMA) could only achieve a maximum of 90° magnetization rotation (from out of plane to in-plane). In our proposed scheme, core reversal can be achieved without requiring any external field. Furthermore, we can switch reversibly between two skyrmion states and two ferromagnetic states, i.e. skyrmion states with the magnetization of the core pointing down/up and periphery pointing up/down, and ferromagnetic states with magnetization pointing up/down, by sequential increase and decrease of the perpendicular magnetic anisotropy.

Electric field control of magnetization reversal offers an extremely energy efficient route for manipulating magnetisation. VCMA^25^,^26^,^27^, strain^28^,^29^,^30^, magnetoelectric switching using antiferromagnetic layer coupled to a ferromagnet^31^,^32^ are techniques that offer electric field control of magnetization in nanomagnets. In this paper, we show with rigorous micromagnetic simulation that a skyrmion core can be reversed solely by modulating the perpendicular anisotropy with an electric field, i.e. without any external magnetic field. This utilizes the dependence of the anisotropy value in the ferromagnet/oxide interface (which originates from the overlap between oxygen’s p_z_ and ferromagnet’s hybridized d_xz_ orbital^33^) on the electron density^34^. We use this voltage control of magnetic anisotropy (VCMA) technique to create and annihilate skyrmions and as well as to achieve core reversal and switching between ferromagnet and skyrmion states. Such electric field control of topologically stable skyrmions can create a new avenue towards implementing energy efficient nanomagnetic computing.

## Result

**Figure 1:**
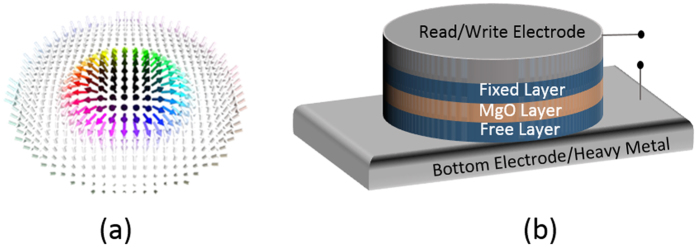
(**a**) Skyrmion (**b**) MTJ structure (variants of the readout schemes in the supplement).

**Figure 2:**
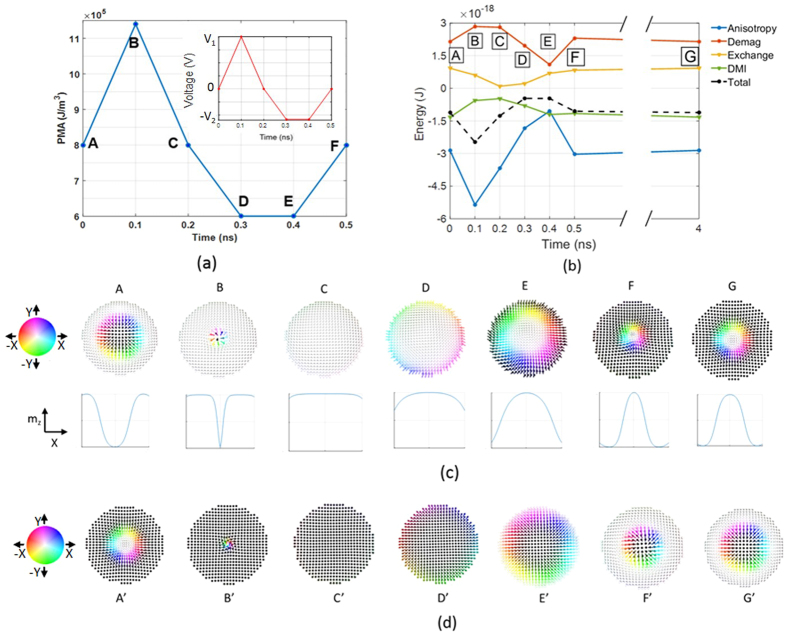
(**a**) Anisotropy energy density and voltage vs time, we considered the rise time and fall time of the electric field to be 100 ps (to charge the capacitive MgO layer). This is typically idealized as a trapezoidal shaped voltage pulse with a dwell time between the rise and fall. However, a tent like (triangular) positive pulse is used to show that skyrmion-ferromagnetic transition can occur as fast as 0.2 ns. In other words, we can immediately remove the electric field once it reaches peak value. We could also use a usual symmetrical trapezoidal shaped positive pulse without affecting the switching physics. (**b**) Energies of different magnetic states at corresponding discrete point in time during the switching process (connecting lines between two points are for ease of visualization and do not represent actual energies as a function of time between these points), (**c**) spin states at different time and associated magnetization component in the z-direction of different points along the diameter, (**d**) Different magnetic sates during the reversal of a skyrmion with upward core.

We simulate the magnetization dynamics in a perpendicular anisotropy CoFeB/MgO/CoFeB MTJ structure shown in Fig. 1(b) to demonstrate skyrmion core reversal. The bottom CoFeB layer is the free layer which is chosen to be a nanodisk with radius of 80 nm and thickness of 1 nm. The reversal of the skyrmionic state is achieved through modulation of the perpendicular magnetic anisotropy by applying an electrical voltage. Modulation of the PMA initiates a change in the orientation of the spins and ultimately the equilibrium spin configuration is determined by minimizing the total energy of the system which includes exchange energy, DMI energy, magnetic anisotropy energy and demagnetisation energy. We note that the micromagnetic simulation describes the evolution of the magnetic configuration with time to reach this local minimum. The reversal is a two-step process. The voltage profile and anisotropy energy density change with time, the magnetic energies of the system at various states and configurations of different magnetic states visited during the switching process are shown in Fig. 2(a–d) respectively.

We start with a skyrmion whose core points down (Fig. 2(c), state A). In the first step, a positive voltage is applied to the skyrmion which strengthens the perpendicular anisotropy. This forces more spins to point in the direction perpendicular to the x-y plane (i.e. in the direction ±z) to reduce the anisotropy energy. Minimization of curvature energy cost of the circular domain wall (i.e. the spin spiral) demands stabilization of a skyrmion with smaller core radius when PMA is increased^35^.

As a result, the diameter of the skyrmion core keeps reducing with increasing PMA (Fig. 2(c), state B). This makes +z direction the preferred direction among the two possible perpendicular spin orientations (±z). Allowing the spins to relax under this condition would transform the magnetization to a complete ferromagnetic state. However, once a sufficient number of spins are pointing in the downward (−z) direction (very small core diameter as can be seen in Fig. 2(c), state B), exchange interaction can drive rest of the core spins downward and thus a ferromagnetic state can be achieved while reducing the applied voltage to zero (Fig. 2(c), state C). Increase in the DMI and demagnetization energy due to this transformation (from state A to state C) is compensated by the reduction in anisotropy and exchange energy as shown in Fig. 2(b). This ferromagnetic state is also stable (similar to the skyrmionic state A) and this is what makes it non-volatile. Note that, spins at the edge of a skyrmion confined in a nanodisk tilt so that they have a magnetization component along the x-y plane^35^. This can be seen by observing magnetization component in the z-direction of different points along the diameter in Fig. 2(c). Therefore, the geometric edge could enable continuous annihilation.

In the next step, a negative voltage is applied to lower the perpendicular anisotropy. When the perpendicular anisotropy is made sufficiently low by applying a large enough negative voltage, the DMI and demagnetization energies become dominant. The spins then rearrange themselves in this altered energy landscape and transforms from the complete ferromagnetic state to an incomplete skyrmion state as shown in Fig. 2(c), state D. In this state, the spins in the core point up (+z) and the spins in the periphery are tilted downward (−z). Under these conditions, the spins finally stabilizes as shown in Fig. 2(c), state E, forming an incomplete skyrmion with skyrmion number between 0.5 and 1. The tilting starts at the periphery of the disk because this results in a smaller penalty in terms of exchange energy than the tilting of the spins in the core. Finally, the applied voltage is removed and the zero bias PMA is restored. The spins in the periphery of the nanodisk now rotates completely to the –z direction and the spins in the core retains their upward (+z) magnetization direction. As a result, a skyrmion state with core pointing up is formed as shown in Fig. 2(c), state F. The skyrmion formed in state F is not at equilibrium but can reach equilibrium without any external bias after some time as shown in Fig. 2(c), state G. This is also non-volatile. Hence, we have bistable skyrmionic state “0” and “1”. A similar voltage pulse can be applied immediately to the skyrmion in state F to switch to the initial magnetic state. Transition from state A to state F takes 0.5 ns. Therefore a memory device with speed of 2 GHz can be realized.

We note that each equilibrium configuration (A, C, G) was attained by forming a magnetic configuration that corresponds to a local energy minimum closest to its prior state, i.e. the state from which this system evolves, and separated from other local minima by an energy barrier. Thus, when the system evolves from a state stabilized by high PMA due to VCMA with a positive voltage, it settles to the ferromagnetic state when the VCMA is reduced to zero. But, when the system evolves from a state stabilized by low PMA due to VCMA with a negative voltage, it settles to the skyrmion state when the VCMA is reduced to zero. But it cannot spontaneously switch between the skyrmion and ferromagnetic state due to the energy barrier separating them.

**Figure 3:**
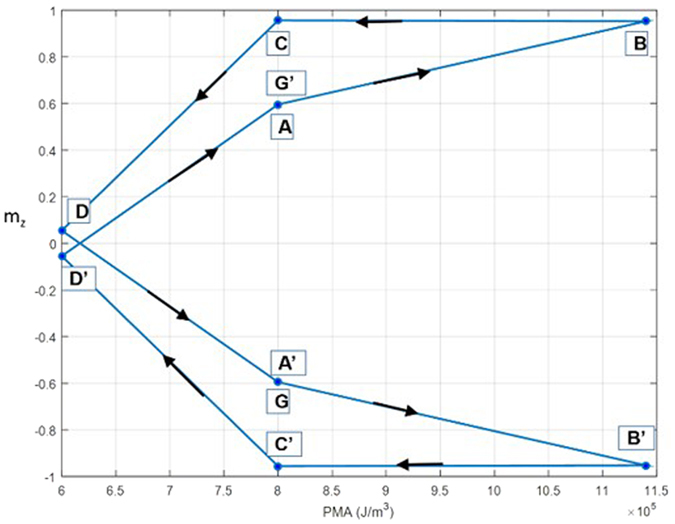
Normalized perpendicular magnetization (m_z_) vs. PMA.

Switching of a skyrmion with upward core spins and downward periphery spins to a skyrmion with downward core spins and upward periphery spins can be achieved by applying the same voltage pulse as shown Fig. 2(a). The transition through the various magnetic states (from A’ to G’) during this switching process is shown in Fig. 2(d). We note that this electrically controlled skyrmion core reversal is deterministic. With a sufficiently long positive (negative) voltage pulse, skyrmion-FM (FM-skyrmion) transition probability does not rely on precise pulse withdrawal as these states are stable and separated by an energy barrier. Consequently, reversible switching between all four states (two skyrmion and two ferromagnetic) is possible as can be seen in Fig. 3. The ability to toggle between the possible states makes this device a viable memory element.

## Discussion

In summary, our simulations have demonstrated the use of voltage controlled magnetic anisotropy for core reversal of a magnetic skyrmion, skyrmion mediated ferromagnetic state reversal and switching between skyrmion and ferromagnet states without requiring any bias magnetic field. More involved simulation that include thermal noise could establish the room temperature stability of various states and error involved in switching between these states, but is beyond the scope of this paper. By integrating an MTJ, reading capability of the different magnetic states can be achieved in the manner of Fig. 1(b). Two additional reading/writing mechanism have been proposed and discussed in the supplementary material of this paper.

We can estimate the energy dissipated in switching between the skyrmions states as follows: the modulation of the interface anisotropy energy is given by J_sa_ = J_0_ + *a*E, where *a*, E and J_0_ are respectively the coefficient of electric field control of magnetic anisotropy, the applied electric field and the interface anisotropy energy at zero bias field. Now, coefficient of electric field control of magnetic anisotropy is defined as, 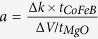, where ∆k is the change in anisotropy energy density, ∆V is the applied voltage, t_CoFeB_ and t_MgO_ are the respective thickness of CoFeB and MgO layer. Reported value of “*a*” is ≈100 *μJ*/*m*^2^ per V/nm with appropriate iridium buffer^36^. Thus, with a 1 nm thick free layer and 1 nm thick MgO layer, 1 × 10^5^ *J*/*m*^3^ change in the anisotropy energy density can be obtained per volt. Note that a thinner fixed layer would provide large PMA and ensure the magnetization of this fixed layer is not affected by the voltage applied. The required maximum and minimum PMA can achieved by applying electrical voltages of V_1_ = 3.4 V and V_2_ = −2 V respectively for the proposed device configuration. These values translate into an energy dissipation of ≈2.4 fJ per switching cycle at a switching speed of 2 GHz if all the energy required to charge the capacitive MgO layer (relative permittivity ≈ 7 [Ref. 37], thickness ≈ 1 nm, diameter ≈ 80 nm) is ultimately dissipated. Insertion of a Hafnium (Hf) monolayer between free and MgO layer can increase “*a*” by 5.2 times^38^. Such optimization can reduce energy dissipation to only ≈90 aJ which is five times less than the energy dissipated to switch a conventional CMOS device^39^ of comparable speed. The diameter of the nanodisk forming the free layer can be scaled down to 40 nm to further reduce the energy dissipation. Furthermore, an advantage of the nanomagnetic element is its non-volatility. Moreover, substantial reduction of energy dissipation may be achieved by lowering the electric field needed for the switching process if the coefficient of anisotropy energy change (*a*) is enhanced in future materials/interfaces. Moreover, we can switch between states in a few nanoseconds, which is competitive for computing applications, particularly given low energy dissipation and non-volatility.

These theoretical results could stimulate experimental work on switching fixed skyrmions with voltage controlled magnetic anisotropy, novel interfaces with higher coefficient of anisotropy energy change (*a*) and lead to implementation of energy efficient memory devices based on skyrmion core reversal or ferromagnetic state reversal (via an intermediate skyrmion state), and Boolean and non-Boolean MTJ based computing in the manner of refs 40,41 respectively.

## Methods

Simulations were performed using the micromagnetic simulation software-Mumax^42^. Our geometry was discretized into 1 × 1 × 1 nm^3^ cells. The change in uniaxial anisotropy constant is realized by modulating the electric field.

In the MuMax framework^42^, the magnetization dynamics is simulated using the Landau-Lifshitz-Gilbert (LLG) equation:


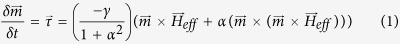


where *m* is the reduced magnetization (M/M_s_), M_s_ is the saturation magnetization, *γ* is the gyromagnetic ratio and *α* is the Gilbert damping coefficient. The quantity *H*_*eff*_ is the effective magnetic field which is given by,


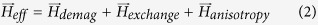


here, 
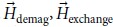
 and 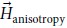 are the effective field due to demagnetization energy, the effective field due to Heisenberg exchange coupling and DMI interaction, and the effective field due to the perpendicular anisotropy evaluated in the MuMax framework in the manner described in ref. 42.

The fixed layer can be designed to be thinner to have much higher PMA which will ensure a minimal effect of changing PMA. In other words the PMA in this fixed layer can be designed to be strong so that any voltage induced change of magnetic anisotropy will not perturb the magnetization direction of this fixed layer. Additionally, the coefficient of electric field control of magnetic anisotropy in the fixed layer can be tailored to be low. For example, one such method could be not inserting the Hafnium monolayer between the MgO and the fixed layer which will make the coefficient of electric field control of magnetic anisotropy smaller in the fixed layer. Furthermore, one can use a synthetic antiferromagnetic (SAF)^43^ layer to increase magnetic stability of the fixed layer and electric field induced magnetization rotation in the fixed layer will be further minimized. Hence, we ignore anti-symmetric modification effects in our model. The synthetic antiferromagnetic (SAF) layer offsets the dipolar interaction between the fixed and the free layer. Hence, we also ignore dipolar effects in our model.

A recent study reported modification of exchange stiffness by applying Electric field^44^ which is not considered in our model. However, a positive (negative) electric field will increase (decrease) the exchange stiffness which will enable easy transformation from skyrmion to ferromagnetic (ferromagnetic to skyrmion) state which can be understood from the energy profile plotted in Fig. 2(b). We have simulated scenarios considering electric field induced modification of exchange stiffness and verified that switching occurs at lower electric field. Hence, the voltage estimates we present are conservative.

Typical parameters for the CoFeB layer are listed in Table 1^15^,^45^. (This is for Co_20_Fe_60_B_20_).

## Additional Information

**How to cite this article**: Bhattacharya, D. *et al*. Voltage controlled core reversal of fixed magnetic skyrmions without a magnetic field. *Sci. Rep.* **6**, 31272; doi: 10.1038/srep31272 (2016).

## Supplementary Material

Supplementary Information

## Figures and Tables

**Table 1:** Typical parameters of CoFeB layer used in our simulations.

Parameters	Value
Saturation Magnetization (M_s_)	1 × 10^6 ^A/m
Exchange Constant (A)	2 × 10^−11 ^J/m
Perpendicular Anisotropy Constant (K_u_)	8 × 10^5 ^J/m^3^
Gilbert Damping (*α*)	0.03
DMI Parameter (D)	3 mJ/m^2^
