# Supplementary material for: Voltage controlled core reversal of fixed magnetic skyrmions without a magnetic field
Source: Sci Rep. 2016 Aug 10;6:31272. doi: 10.1038/srep31272 (PMC4978972; doi:10.1038/srep31272)
Supplement: Supplementary Information [file srep31272-s1.pdf]

SUPPLEMENTARY INFORMATION

Voltage controlled core reversal of fixed magnetic skyrmions without a magnetic field

Dhritiman Bhattacharya[1], Md Mamun Al-Rashid[1,2] and Jayasimha Atulasimha[1, 2 *]

[1]*Department of Mechanical and Nuclear Engineering, Virginia Commonwealth University, Richmond, VA 23284, USA*
[2]*Department of Electrical and Computer Engineering, Virginia Commonwealth University, Richmond, VA 23284, USA*
* Corresponding author: jatulasimha@vcu.edu

Read out schemes: The device proposed in Figure 1(b) of the main paper allows simple and direct reading/writing of the various states (i.e. two skyrmionic and two ferromagnetic state). However, the magnetizations in the core and the periphery of the skyrmionic states are anti-parallel and as a result, this scheme suffers in terms of low magnetoresistance ratio while reading these states. This issue can be addressed by designing the electrodes to only read either the core or the periphery of the nanodisk. A 3 terminal device configuration is shown in Figure S1 (a) where a reading electrode of 15 nm diameter is delineated on top of the fixed layer which also has a diameter of 15 nm. Such a small MTJ hard layer can be fabricated without losing thermal stability [S1]. An annular electrode is used for writing (i.e. modifying the PMA) with 20 nm inner diameter and 80 nm outer diameter. This scheme is suitable to read the core of the nanodisk. This configuration also provides separation between the read and write operation, potentially providing improved reliability. Another alternative is to read the magnetization state of the periphery. Figure S1 (b) shows a device structure which fulfills this purpose. This 2 terminal structure gets rid of the small read electrode of the previous 3 terminal device and uses the annular electrode (20 nm inner diameter and 80 nm outer diameter) for both reading and writing. The physics behind the reversal by applying electric field only in the peripheral region does not differ from the one described in the main paper. Different magnetic states visited in response to perpendicular energy density change with voltage (Figure S2) applied in the peripheral region only are shown in Figure S3. Note that we need a slightly higher positive voltage pulse in the latter two methods to switch the skyrmion.

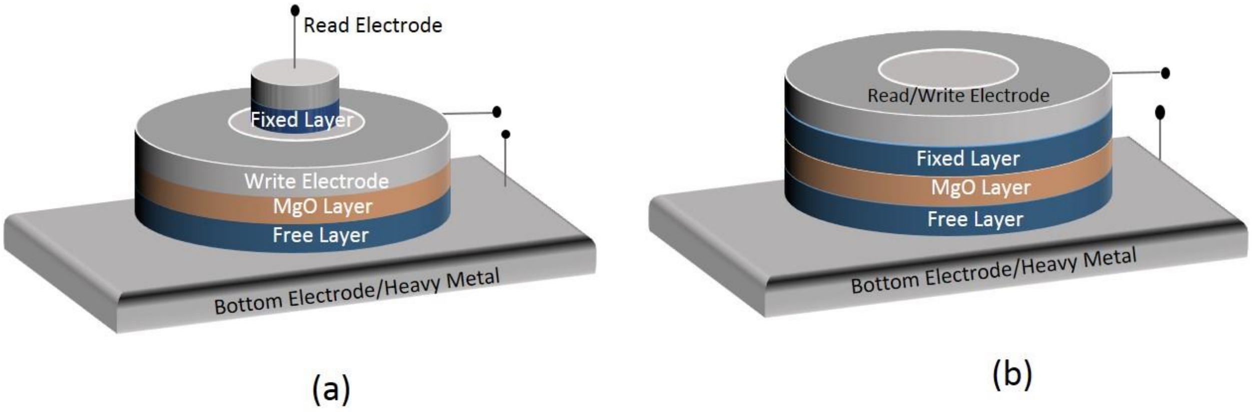

FIG. S1. MTJ structure to read (a) only the skyrmion core and (b) only the skyrmion periphery.

Other ways to control magnetic anisotropy: In addition to directed voltage control of magnetic anisotropy one could modulate the magnetic anisotropy of magnetostrictive nanomagnets with acoustic waves or voltage generated strain in a piezoelectric layer in elastic contact with the nanomagnet. However, assuming optimistically that ~100 MPa could be generated in a Terfenol-D nanomagnet with magnetostriciton ~ 1000 ppm[S2] the effective change in magnetic anisotropy energy density ~10^5 J/m^3 can

be achieved. This is about one order of magnitude smaller than the required change in anisotropy. Hence, strain mediated switching of skyrmion states could be feasible only if materials that have much larger magnetoelastic coupling and sufficient DMI (when interfaced with appropriate materials] to form skyrmions are developed.

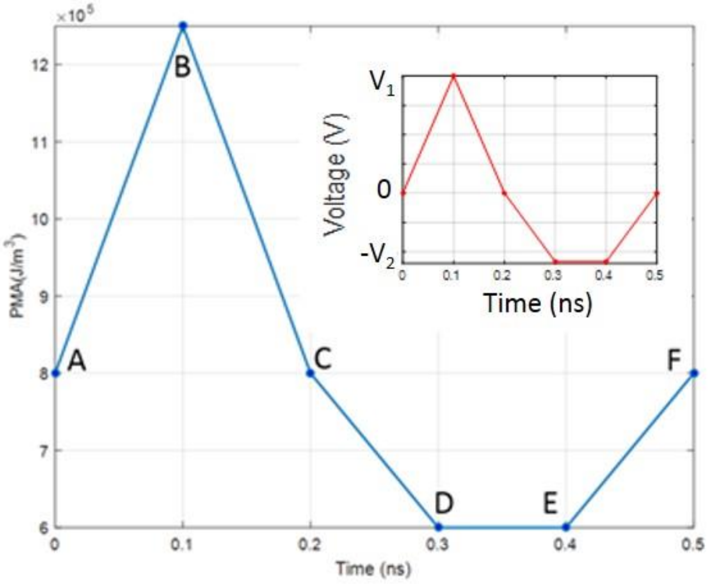

FIG. S2. Anisotropy energy density and voltage vs. time for switching using the outer ring only.

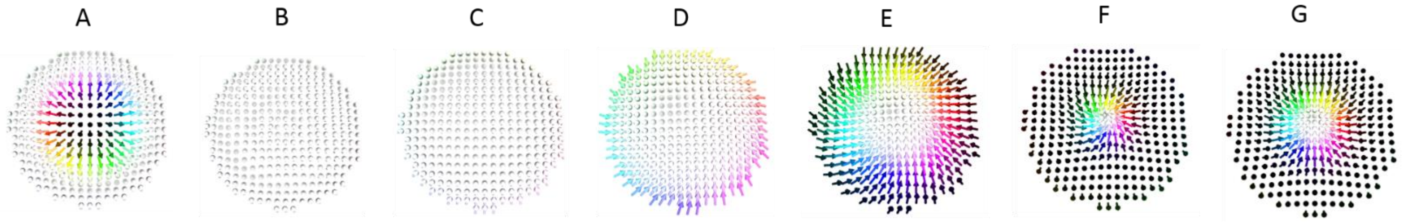

FIG. S3. Spin states visited at different times during core reversal using outer ring only.

REFERENCES

[S1] Kim et al, Verification on the extreme scalability of STT-MRAM without loss of thermal stability below 15 nm MTJ cell, Symp. VLSI Technol., Dig. Tech. Papers, Jun. 2014, pp. 1–2.

[S2] K. Ried, M. Schnell, F. Schatz, M. Hirscher , B. Ludescher, W. Sigle and H. Kronmuller Phys. Status Solidi a 167 195, 1998.
